# Predicting Post-surgery Discharge Time in Pediatric Patients Using Machine Learning

**DOI:** 10.37825/2239-9747.1055

**Published:** 2024-07-18

**Authors:** Marco Cascella, Cosimo Guerra, Atanas G. Atanasov, Maria G. Calevo, Ornella Piazza, Alessandro Vittori, Alessandro Simonini

**Affiliations:** a Anesthesia and Pain Medicine, Department of Medicine, Surgery and Dentistry “Scuola Medica Salernitana”, University of Salerno, Baronissi, 84081, Italy; b Institute of Genetics and Animal Biotechnology of the Polish Academy of Sciences, Jastrzebiec, 05-552, Magdalenka, Poland; c Laboratory of Natural Products and Medicinal Chemistry (LNPMC), Center for Global Health Research, Saveetha Medical College and Hospital, Saveetha Institute of Medical and Technical Sciences (SIMATS), Thandalam, Chennai, India; d Ludwig Boltzmann Institute Digital Health and Patient Safety, Medical University of Vienna, Spitalgasse 23, 1090, Vienna, Austria; e Epidemiology and Biostatistics Unit, Scientific Direction, IRCCS Istituto Giannina Gaslini, Genoa, Italy; f Department of Anesthesia and Critical Care, ARCO ROMA, Ospedale Pediatrico Bambino Gesù IRCCS, Piazza S. Onofrio 4, 00165, Rome, Italy; g Pediatric Anesthesia and Intensive Care Unit AOU delle Marche, Salesi Children’s Hospital, 60121, Ancona, Italy

**Keywords:** Machine learning, Artificial intelligence, Tonsillectomy, Random forest, Postoperative nausea and vomiting

## Abstract

**Background:**

Prolonged hospital stays after pediatric surgeries, such as tonsillectomy and adenoidectomy, pose significant concerns regarding cost and patient care. Dissecting the determinants of extended hospitalization is crucial for optimizing postoperative care and resource allocation.

**Objective:**

This study aims to utilize machine learning (ML) techniques to predict post-surgery discharge times in pediatric patients and identify key variables influencing hospital stays.

**Methods:**

The study analyzed data from 423 children who underwent tonsillectomy and/or adenoidectomy at the IRCCS Istituto Giannina Gaslini, Genoa, Italy. Variables included demographic factors, anesthesia-related details, and postoperative events. Preprocessing involved handling missing values, detecting outliers, and converting categorical variables to numerical classes. Univariate statistical analyses identified features correlated with discharge time. Four ML algorithms—Random Forest (RF), Logistic Regression, RUSBoost, and AdaBoost—were trained and evaluated using stratified 10-fold cross-validation.

**Results:**

Significant predictors of delayed discharge included postoperative nausea and vomiting (PONV), continuous infusion of dexmedetomidine, fentanyl use, pain during discharge, and extubation time. The best-performing model, AdaBoost, demonstrated high accuracy and reliable prediction capabilities, with strong performance metrics across all evaluation criteria.

**Conclusion:**

ML models can effectively predict discharge times and highlight critical factors impacting prolonged hospitalization. These insights can enhance postoperative care strategies and resource management in pediatric surgical settings. Future research should explore integrating these predictive models into clinical practice for real-time decision support.

## 1. Introduction

Efficient management of hospital resources is crucial for optimizing patient care and ensuring the sustainability of healthcare systems. Consequently, accurate prediction of discharge times can significantly enhance hospital operations by enabling better bed management, staffing, and scheduling, ultimately leading to improved patient outcomes and satisfaction [1].

Prolonged hospital stays after pediatric surgeries represent a significant concern, both in terms of cost and impact on patient care. The prevalence of extended hospitalizations varies greatly depending on the type of chronic condition and among different children’s hospitals [2]. Multiple variables can affect hospital length of stay [3]. For tonsillectomy and adenoidectomy procedures, research typically examines discharge times from the recovery room. In this setting, common causes of prolonged hospitalization include younger age and postoperative desaturation [4]. Moreover, the use of opioids and upper respiratory infections have been identified as potential predisposing factors [5]. Additionally, postoperative nausea and vomiting (PONV) can also extend hospital stays [6]. On the contrary, postoperative pain, while common in children undergoing tonsillectomy, is a less frequent and controversial cause of prolonged hospitalization [7].

On these premises, understanding the determinants of prolonged hospitalization is of paramount importance for optimizing postoperative care and resource allocation. Given that multiple variables including patient-related factors as well as surgical, anesthesia-related, and postoperative factors can contribute to extended discharge times, an exhaustive investigation of the potential causes underlying prolonged hospital stays is required. In this context, the application of artificial intelligence and machine learning (ML) techniques is a suitable strategy for predicting post-surgery discharge times more accurately [8].

One of the key advantages of using ML in healthcare is its ability to handle large and complex datasets. Traditional methods often rely on manual analysis or statistical approaches, which may overlook subtle patterns or interactions within the data. ML algorithms, on the other hand, can automatically identify relevant features and relationships, leading to more accurate predictions. Briefly, ML models can consider various factors such as patient demographics, medical history, surgical procedure, vital signs, and recovery progress to forecast the optimal time for discharge. These models can adapt and improve over time as they receive more data and feedback, making them increasingly accurate and reliable. Therefore, by leveraging patient data and clinical variables, ML-based strategies can provide insights into the factors influencing recovery trajectories, facilitating more efficient healthcare delivery and better patient outcomes [9,10].

The purpose of the following study is to identify which variables determine the time of discharge and to train a series of algorithms capable of predicting discharge times in pediatric patients undergoing surgical and anesthetic procedures (i.e., one-day surgery).

## 2. Methods

### 2.1. Study population and data collection

The study population consists of 423 children who underwent tonsillectomy and/or adenoidectomy. The primary investigation occurred at the IRCCS Istituto Giannina Gaslini in Genoa, Italy, with approval from the institutional Ethics Committee (protocol number 048/2018) [11]. This dataset encompasses various demographic factors such as age, gender, weight, presence of neurocognitive issues, other comorbidities, occurrence and type of respiratory infections in the 7 days before the surgery, obstructive sleep apnea (OSAS), anesthesia risk (American Society of Anesthesiologists, ASA), and medication usage. Additionally, the collection includes anesthesia-related details including preanesthesia features (drug, dosage), anesthesia induction (drug, dosage), and maintenance (technique, drugs, dosages, fluid therapy), intraoperative events (bradycardia, tachycardia, hypotension, intraoperative movements), surgery duration, and post-surgery/anesthesia emergence variables including extubation time in minutes, occurrences of laryngospasm, desaturation, postoperative nausea and vomiting (PONV), and other adverse events. In this regard, we considered bradycardia as a heart rate (HR) decrease of more than 20% from baseline, and tachycardia an increase of more than 20% from baseline. Hypotension is defined as a systolic blood pressure reduction of more than 20% from baseline, and hypertension a mean arterial pressure increase of more than 20% from baseline. Desaturation refers to a drop in SpO_2_ levels below 90% of the baseline value for over 15 seconds.

Data from the Post-Anesthesia Care Unit (PACU) includes time to full awakening, incidents of emergence delirium (ED), pain assessment using the Face, Legs, Activity, Cry, Consolability Scale (FLACC) or the Numeric Rating Scale (NRS), bradycardia, and desaturation. Postoperative monitoring from PACU to discharge involved recording occurrences of PONV, pain scores, and adherence to discharge timing (within 24 hours). As per the primary investigation, the Pediatric Anesthesia Emergence Delirium (PAED) tool was employed to evaluate ED [12]. During PACU monitoring, the PAED scale was administered thrice at 10-minute intervals by a dedicated nurse, with the highest PAED score among the three assessments considered for data collection purposes. The dataset is accessible at [13].

### 2.2. Preprocessing and exploratory data analysis

Preprocessing, exploratory data analysis, and statistical correlation analyses were conducted using SPSS software version 27. Initially, null values present in the dataset were imputed as “0”, as they represent the absence of the specific variable. Using the Mahalanobis distance method, 27 data points containing outliers were detected [14]. However, we ultimately decided not to remove them, as their exclusion caused a drop in the algorithm’s performance, indicating that they were informative for the prediction task.

The frequencies of categorical variable classes and basic descriptive parameters (range, mean, and standard deviation) for numerical variables were examined. Variables with literal classes were converted to numerical classes.

A new column, representing the patient’s age at the time of surgery, was computed as the difference between the surgery date and the birth date. The columns related to the use of sugammadex and ondansetron were not considered, as they were constant.

A univariate statistical analysis was then performed to examine the correlation between categorical features and discharge time using the Chi-square test (*p* < 0.05) and between continuous features and discharge time using point–biserial correlation. An alpha significance level of 0.05 was selected.

Features that were initially determined to be correlated with discharge time were selected as input features for the algorithm training. Subsequently, the presence of multicollinearity among the selected features was explored.

### 2.3. Predictive modelling

The dataset was divided into a training subset (n = 283 observations) and a test subset (n = 140 observations) by ensuring that the test set contained 33% of total observations. Four algorithms were selected and optimized with a Bayesian search optimization process, an approach that uses stepwise Bayesian Optimization to explore the most promising hyperparameters set in the problem space:

- Random Forest (RF), a commonly used ML algorithm that combines the output of multiple decision tree structures to achieve a single result. For classification tasks, the output of the RF is the class selected by most trees [15].
- Logistic regression (LR) model. It is a multivariable method for modeling the relationship between multiple independent variables and a categorical dependent variable, widely used in medical and clinical research [16].
- RUSBoost, an algorithm for solving the problem of class imbalance. It combines data sampling and boosting, providing a simple and efficient method for improving classification performance when training data is unbalanced [17].
- AdaBoost (adaptive boosting), an ensemble learning algorithm that can be used for classification or regression. It uses multiple iterations to generate a single composite strong learner. During each round of training, a new weak learner is added to the ensemble, and a weighting vector is adjusted to focus on examples that were misclassified in previous rounds. The result is a classifier that has higher accuracy than the classifiers of the weak learners [18].

The models were then evaluated using a stratified 10-fold cross-validation. This is a variation of classical K-fold cross-validation that ensures each fold maintains the same proportion of observations for each target class as the complete dataset. This approach is especially crucial for datasets where one class might be heavily underrepresented.

Several metrics were then used to evaluate the algorithms’ performances [19]:

- Accuracy, defined as the ratio of correctly predicted observations to the total observations. It is the ratio between the number of correct predictions (True Positives and True Negatives) and the total number of predictions. This metric gives a straightforward indication of the model’s overall correctness. If a model has high accuracy, it means that it is making a large number of correct predictions relative to the total number of predictions. However, accuracy may not always be the best indicator of model performance, especially in cases of class imbalance. For example, in a dataset where one class is much more frequent than the other (e.g., 95% of instances are negative and only 5% are positive), a model that always predicts the majority class will have high accuracy but poor performance in identifying the minority class. Thus, accuracy should be considered alongside other metrics like Precision, Recall, and the F1 Score to obtain a more comprehensive evaluation of the model’s performance.
- Precision and Recall. Precision is the ratio of correctly predicted positive observations to the total predicted positives. High precision relates to a low rate of false positives. Recall is the ratio of correctly predicted positive observations to all observations in actual class: high recall relates to a low rate of false negatives.
- F1 Score, defined as the weighted average of Precision and Recall. In other words, it provides a single metric that reflects both the Precision and Recall of a classifier, offering a comprehensive assessment of its performance. By considering both false positives and false negatives, the F1 Score offers a holistic perspective on the model’s ability to correctly classify instances across all classes. Consequently, a higher F1 Score signifies a classifier that achieves a balance between precision and recall, indicating robust performance in identifying relevant instances while minimizing misclassifications.
- Area Under the Receiver Operating Characteristic Curve (AUC-ROC): The ROC curve is a graphical representation of the contrast between true positive rates and false positive rates at various thresholds. The AUC represents a degree of separability achieved by the model. A higher AUC, approaching 1, value correlates with a model better capable of distinguishing between the positive and negative classes.
- Confusion Matrix: A confusion matrix is a table that is often used to describe the performance of a classification model on a set of test data for which the true values are known. It provides insights not just into the errors being made by a classifier but also the types of errors that are being made.

The selection, optimization, cross-validation, and evaluation of the algorithm were performed in Python (version 3.10.6) using the scikit-learn package.

## 3. Results

### 3.1. Exploratory data analysis results

Frequencies of classes for categorial variables are shown in Table 1.

Results for continuous variables are reported in Table 2.

The average age at the time of surgery was 4.14 ± 1.45 standard deviation, with 56.5% male and 43.5% female. An amount of 31.44% had a history of OSAS. A pre-anesthesia phase was conducted in 93.62% of the children using midazolam (95.5%) and/or ketamine (95.5%). Most patients (97%) underwent inhalation anesthesia. During the maintenance phase, 49.79% and 29.79% were administered fentanyl and clonidine respectively. Concerning complications, 6.62%, 8.27%, 6.62%, 3.07%, and 3.31% developed bradycardia, tachycardia, hypotension, hypertension, and intraoperative movements, respectively. Upon awakening, 15.84% developed oxygen desaturation and 16.02% laryngospasm. We calculated that 25.77% experienced delirium and 4.97% pain. Regarding discharge time, 5.44% of patients were discharged after 24 hours.

From the univariate statistical analysis, eight variables were identified as most correlated to Discharge time (Table 3).

Asignificant but weak negative association resulted in the use of fentanyl and being discharged within 24 hours (*p* < 0.001, *χ*2 = 19.382, *ϕ* = −0.214). Moreover, there is a significant negative moderate association between continuous infusion of dexmedetomidine and discharge within 24 hours (*p* < 0.001, *χ*2 = 53.459, *ϕ* = −0.355), independently of the dose used (*p* = 0.056) and a significant negative but negligible association between the presence of other adverse events and discharge within 24 hours (*p* = 0.001, *χ*2 = 12.018, *ϕ* = −0.169). Additionally, there is a strong negative association between the presence of PONV and timely discharge within 24 hours, indicating that patients experiencing PONV are less likely to be discharged within 24 hours compared to those who do not suffer from it (*p* < 0.001, *χ*2 = 108.234, *ϕ* = −0.506). There is also a significant weak negative association between pain and discharge within 24 hours (*p* < 0.001, *χ*2 = 17.230, *ϕ* = −0.202). Moreover, the minutes spent for extubation are significantly correlated with being discharged later than 24 hours (*p* < 0.001, *r* = −0.312).

For patients receiving continuous dexmedetomidine infusion, the odds of on-time discharge were significantly reduced (OR = 0.092, 95% CI [0.061, 0.158]), while the odds for discharge after 24 hours were increased (OR = 1.504, 95% CI [1.152, 1.964]). Fentanyl use was associated with decreased odds of on-time discharge (OR = 0.112, 95% CI [0.101, 0.374]) and a marginal increase in odds for discharge after 24 hours (OR = 1.059, 95% CI [1.031, 1.173]). Experiencing adverse events resulted in significantly lower odds of on-time discharge (OR = 0.161, 95% CI [0.129, 0.494]). The odds of being discharged after 24 hours showed a tendency to increase, although not statistically significant (OR = 1.359, 95% CI [0.905, 2.011]). Patients reporting pain during discharge were less likely to be discharged on time (OR = 0.216, 95% CI [0.099, 0.470]). However, there was a notable increase in the likelihood of being discharged after 24 hours (OR = 1.146, 95% CI [1.034, 1.270]).

The occurrence of PONV drastically reduced the odds of on-time discharge (OR = 0.040, 95% CI [0.017, 0.096]) and significantly increased the odds of discharge after 24 hours (OR = 1.628, 95% CI [1.259, 2.078]). Oxygen desaturation at the time of discharge decreased the likelihood of on-time discharge (OR = 0.298, 95% CI [0.110, 0.811]). The odds for discharge beyond 24 hours showed a slight increase but were not conclusively significant (OR = 1.134, 95% CI [0.954, 1.347]).

To ensure the absence of multicollinearity between predictor features, the variance inflation factor (VIF) has been calculated. Two highly correlated features (VIF >5) were found (Table 4).

### 3.2. Predictive modeling results

The performance metrics of the ML models are summarized in Table 5.

**Fig. 1:**
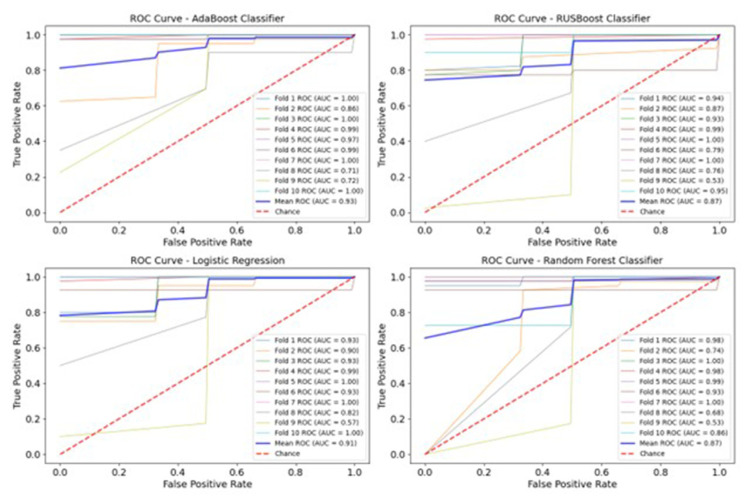
ROC curves for the four algorithms including the single AUC values for each fold.

The AdaBoost algorithm outperformances the other models in at least 3 out of the total 6 metrics measured; in particular, it exhibits a higher ROC-AUC, demonstrating that, on average, the algorithm efficiently distinguishes between the two classes and accurately classifies them in most cases (Fig. 1).

**Fig. 2:**
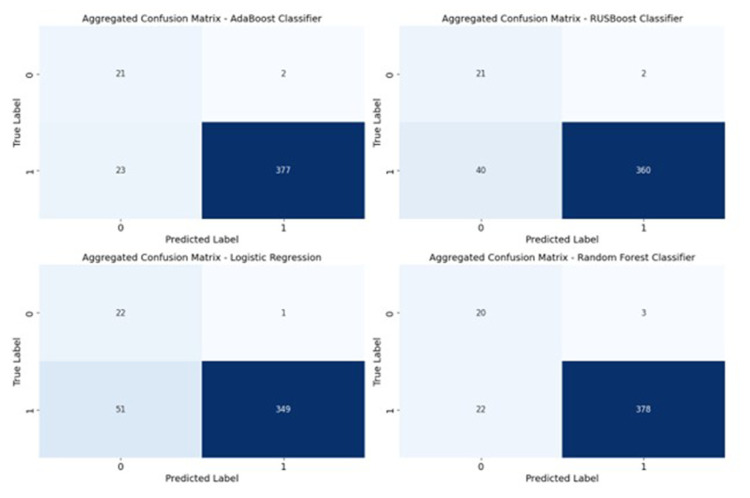
Aggregated confusion matrices of the four algorithms. 0: Discharge after 24 hours, 1: Discharge in 24 hours.

The aggregated confusion matrices were calculated by summing the true positives, true negatives, false positives, and false negatives across each fold. Examination of these matrices reveals that AdaBoost accurately predicted 376 out of 400 instances of discharges occurring within 24 hours, and correctly identified 21 instances as not occurring within 24 hours. Additionally, there were only 2 false positives and 23 false negatives noted (Fig. 2).

**Fig. 3:**
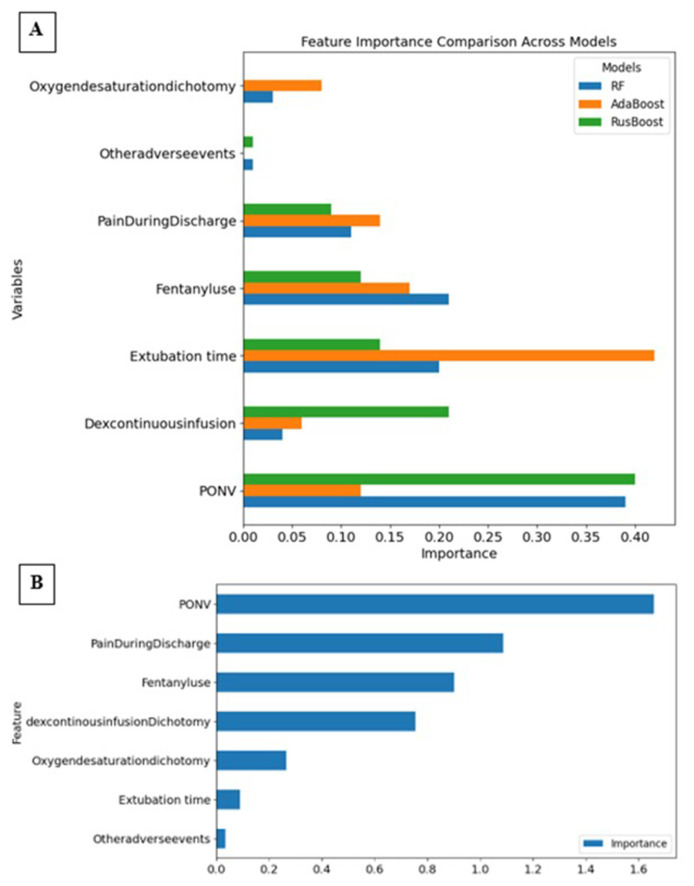
Bar plots representing the features’ importance. A = Ensemble models; B = Logistic Regressor. Abbreviation: RF, Random Forest.

Subsequently, we examined the importance of the various features across the models. Feature importance for the ensemble learning models (AdaBoost, RF, RusBoost) was obtained using the *feature_ importances_* attribute. For the LR model, we calculated the average importance based on the absolute values of the weights of each feature derived from the trained model. Feature importances are shown in Fig. 3 and Table 6.

In the RF model, PONV emerged as the most critical feature with an importance score of 0.39, indicating its substantial impact on discharge prediction. Fentanyl use and Extubation time also had high importance scores of 0.21 and 0.20, respectively, reflecting their significant roles in predicting discharge times. Pain during discharge and oxygen desaturation (as a dichotomy), with importance scores of 0.11 and 0.03 respectively, were less influential but still notable.

The AdaBoost model highlighted extubation time as the most important feature with an importance score of 0.42, suggesting that AdaBoost is particularly sensitive to this variable. PONV and fentanyl use were also important, with scores of 0.12 and 0.17 respectively, though to a lesser extent compared to RF. The importance of pain during discharge and oxygen desaturation dichotomy in AdaBoost was similar to their importance in the RF model, with scores of 0.14 and 0.08 respectively.

In the RusBoost model, PONV once again ranked highest with an importance score of 0.40, consistent with its critical role across models. Dexmedetomidine continuous infusion and fentanyl use had considerable importance, with scores of 0.21 and 0.12 respectively, highlighting their relevance in this model. Pain during discharge and oxygen desaturation showed varied levels of influence, with scores of 0.09 and 0.0 respectively.

The LR model also highlighted PONV as the most important feature, with a notably high average importance score. Pain during discharge, fentanyl use, and dexmedetomidine continuous infusion were also significant, reflecting their strong associations with discharge times. Oxygen desaturation dichotomy and extubation time had moderate importance, indicating their roles in the model, albeit less prominent compared to the ensemble models.

## 4. Discussion

Discharge time is a critical factor in the process of one-day surgery. In this study, 5.44% of the children were discharged after 24 hours, thus experiencing a longer hospital stay. Of these children, 73.9% exhibited the onset of PONV, 43.5% were under continuous dexmedetomidine infusion, 47.8% manifested pain during the discharge process, and 13% developed other side effects. Additionally, 87% received fentanyl during the maintenance phase of anesthesia.

The univariate analysis showed that children who received intraoperative fentanyl were less likely to be discharged on schedule compared to those who did not use that opioid. Moreover, patients who experienced pain or PONV were less likely to be discharged within the expected timeframe compared to those without pain. This result confirms that pain management has a significant impact on the discharge process [20–22].

The onset of PONV has been identified in various studies as one of the main factors contributing to a significant percentage of unplanned readmissions after surgery, along with the onset of side effects induced by the use of anesthetics. PONV is one of the main side effects of general anesthesia. This complication affects patient satisfaction, increases time-to-discharge from the PACU or hospital, and may even be present after discharge from the hospital, leading to rehospitalization [23]. PONV is a common complication following surgical procedures, influenced by several risk factors, many of which are inherent and not modifiable. Not modifiable factors contributing to PONV include gender, history of PONV or motion sickness, age, and type of surgery [24]. Nevertheless, in the realm of anesthesia and postoperative care, some risk factors can be adjusted to minimize the risk of PONV [25]. A critical review of the literature and practice reveals that the management of intra-operative opioids, specifically fentanyl, plays a significant role in PONV outcomes. The research highlighted that an opioid-free total intravenous anesthesia (TIVA) approach, which utilizes drugs such as propofol, ketamine, and dexmedetomidine instead of traditional opioids and volatile anesthetics, significantly reduced the incidence and severity of PONV [26]. Further studies have explored the specific impact of fentanyl when used intra-operatively. The results were striking, with the fentanyl group experiencing a higher demand for postoperative analgesia and a significantly greater incidence of PONV requiring medical intervention [27,28]. In our analysis, we found that, among the children administered fentanyl during the maintenance phase of anesthesia, 16.6% exhibited PONV in the transition from the PACU to discharge.

Dexmedetomidine is a useful anesthetic adjunct, increasingly popular during pediatric surgery and procedural sedation [29]. Notably, in our study, the infusion of dexmedetomidine was correlated with a longer hospital stay. This is in contrast with meta-analysis indicating that this medication reduced postoperative pain and postoperative complications such as delirium and desaturation [30]. However, other studies support our findings. For instance, West et al. [31] found evidence of a slight association between intraoperative dexmedetomidine and the duration of recovery from propofol anesthesia in children. They identified a potential dose relationship, indicating approximately a 15-minute delay in recovery for each μg/kg of dexmedetomidine administered. Given this uncertainty, further research on the effects of dexmedetomidine in pediatric anesthesia should better explore this relationship.

Concerning the ML-based predictive modeling, four classification algorithms were selected and evaluated individually, and after cross-validation, the model with the best performance based on the metrics used (ROC- AUC, Precision-recall curve, precision, recall, accuracy, F1-score) was chosen. Our investigations demonstrated that utilizing an AdaBoost classifier enables accurate and precise classification of pediatric patients’ discharge times based on seven significant features. Briefly, the ML analysis demonstrates that PONV, extubation time, fentanyl use, and pain during discharge are the most influential features across the models. Importantly, each model provided a unique perspective on feature importance due to their different underlying algorithms and sensitivity to various predictors as ensemble models like RF, AdaBoost, and RusBoost tend to capture complex interactions between features, while LR highlights linear relationships. Overall, although the RF model showed better recall and F1 Score, the AdaBoost model demonstrated superior predictive capabilities with high accuracy, and AUC-ROC values, indicating robust performance across various evaluation criteria. The model’s confusion matrix further confirmed its reliability, with a high number of true positives and true negatives. This may be attributed to its ensemble nature, which allows the algorithm to effectively leverage multiple weak learners, build a robust predictive model, and capture complex relationships within the data [32].

The descriptive analysis and ML analysis in this study are closely aligned because they both utilize the same data and variables, with descriptive analysis providing foundational insights that guide and validate the ML models. This alignment ensures that the models are both accurate and interpretable, ultimately supporting better clinical outcomes. In other words, while both descriptive and ML analyses focus on the same data and variables, they offer distinct interpretative perspectives. Descriptive analysis provides foundational insights, outlining patterns and associations within the dataset, while ML analysis leverages these insights to predict outcomes and patterns unseen in the data. Consequently, while their shared focus on variables like PONV, fentanyl use, and pain during discharge, the interpretation of results differs; descriptive analysis elucidates current trends, while ML analysis forecasts future scenarios [33]. This underscores the complementary nature of these analytical approaches, which together provide comprehensive insights for improving clinical practice and patient outcomes [34,35]. Future research should focus on integrating these predictive models into clinical practice and exploring their impact on patient outcomes and healthcare efficiency. Nevertheless, integrating these predictive models into real-time clinical workflows presents challenges, including the need for seamless integration with electronic health records (EHR), user-friendly interfaces for clinicians, and continuous model updating and validation.

### 4.1. Study limitations

The relatively small and geographically limited sample size may not be representative of broader pediatric populations undergoing tonsillectomy and adenoidectomy in different regions or healthcare settings. Therefore, the findings might not be generalizable to all pediatric patients or other types of surgeries. Moreover, the study focused on specific variables related to demographic factors, anesthesia details, and postoperative events. Other potentially relevant factors, such as socioeconomic status, family history, or environmental influences, were not considered and could affect discharge times.

There was a significant imbalance in some of the categorical variables, such as intellectual disability and cardiovascular drug usage, with very few cases in certain categories. This imbalance could affect the performance and robustness of the ML models, particularly in identifying predictors from underrepresented groups. Several methodological approaches can be applied to overcome this issue. For example, this could involve using techniques such as the synthetic minority over-sampling technique (SMOTE) to ensure the ML models are not biased towards the majority class [36]. Finally, the main limitations in the applicability of the model in a clinical setting are related to the relatively low number of general observations and the few instances of children who were discharged after 24 hours (5.75%). More data and additional studies will be needed to corroborate the reliability of this model.

## 5. Conclusions

Predicting postoperative discharge time is a critical factor in optimizing the post-surgery care process and reducing the economic and healthcare costs associated with unexpected extended hospital stays. By implementing ML models, it may be possible to predict whether patients will be discharged within or after 24 hours by identifying modifiable risk factors. Key factors, such as fentanyl use, the occurrence of PONV, and pain during the discharge period, strongly correlate with extended discharge times.

## Figures and Tables

**Table 1:** Class frequencies for categorical variables.

Variable	Frequency (class: frequency, percentage)
Gender	0 (Male): 239, 56.5%
	1 (Female): 184, 43.5%
ASA risk scale	1:211,49,9%
	2: 207,48.9%
	3: 5, 1.2%
Intellectual disability	0 (No): 416, 98.3%
	1 (Yes): 7, 1.7%
Cardiovascular drugs	0 (No): 421, 99.5%
	1 (Yes): 2, 0.5%
Neuropsychiatric therapy	0 (No): 421, 99.5%
	1 (Yes): 2, 0.5%
Chronic diseases	0 (No): 402, 95%
	1 (Yes): 21, 5%
Infections 7 days preoperatively	0 (No): 380, 89.8%
	1 (Yes): 43, 10.2%
Obstructive apnea syndrome	0 (No): 290, 68.6%
	1 (Yes): 133, 31.4%
Preanesthesia	0 (Not administered): 396, 93.6%
	1 (Administered): 27, 6.4%
Midazolam	0 (Used): 402, 95%
	1 (Not used): 21, 5%
Ketamine	0 (Used): 404, 95.5%
	1 (Not used): 19, 4.5%
Parents at anesthesia induction	0 (Not present): 32, 7.6%
	1 (Present): 391, 92.4%
Intravenous anesthesia induction	0 (Not conducted): 411, 97.2%
	1 (Conducted): 12, 2.8%
Inhalation anesthesia induction	0 (Not conducted): 11, 2.6%
	1 (Conducted): 412, 97.4%
Inhalation agents	0 (Not used): 11, 2.6%
	1 (Used): 412, 97.4%
Dexmedetomidine continuous infusion	0 (Not used): 395, 93.4%
	1 (Used): 28, 6.6%
Fentanyl use	0 (No): 242, 57.2%
	1 (Yes): 181, 42.8%
Clonidine use	0 (No): 297, 70.2%
	1 (Yes): 126, 29.8%
Type of Anesthesia	0 (Balanced): 400, 94.6%
	1 (TIVA): 23, 5.4%
Bradycardia	0 (No): 395, 93.4%
	1 (Yes): 28, 6.6%
Tachycardia	0 (No): 388, 91.7%
	1 (Yes): 35, 8.3%
Hypotension	0 (No): 395, 93.4%
	1 (Yes): 28, 6.6%
Hypertension	0 (No): 410, 96.9%
	1 (Yes): 13, 3.1%
Intraoperative movements	0 (No): 409, 96.7%
	1 (Yes): 14, 3.3%
Type of electric knife	0 (Bipolar or RM bipolar): 159, 37.6%
	1 (Cold): 264, 62.4%
Oxygen desaturation	0 (No): 356, 84.2%
	1 (Yes): 67, 15.8%
Laryngospasm	0 (No): 355, 83.9%
	1 (Yes): 68, 16.1%
Other adverse events	0 (No): 413, 97.6%
	1 (Yes): 10, 2.4%
Emergence of delirium	0 (No): 314, 74.2%
	1 (Yes): 109, 25.8%
Delirium scale (PAED 0–10)	• 0: 317, 74.9%
	• 10: 19, 4.5%
	• 11: 3, 0.7%
	• 12: 34, 8%
	• 13: 3, 0.7%
	• 14: 18, 4.3%
	• 15: 7, 1.7%
	• 16: 10, 2.4%
	• 17: 3, 0.7%
	• 18: 4, 0.9%
	• 19: 2, 0.5%
	• 20: 3, 0.7%
Pain after surgery	0 (No): 402, 95%
	1 (Yes): 20, 4.7%
Pain (FLACC)	0: 402, 95%
	1: 20, 4.7%
	6: 1, 0.2%
Bradycardia (in PACU)	0 (No): 399, 94.3%
	1 (Yes): 24, 5.7%
Oxygen desaturation during discharge	0 (No): 398, 94.1%
	1 (Yes): 25, 5.9%
PONV	0 (No): 380, 89.8%
	1 (Yes): 43, 10.2%
Pain during discharge	0 (No): 353, 83.5%
	1 (Yes): 70, 16.5%
Discharge on time (24 hrs)	0 (No): 23, 5.4%
	1 (Yes): 400, 94.6%

**Table 2:** Descriptive statistics for continuous variables.

	N	Minimum	Maximum	Mean	Std. Deviation
Age	423	1	10	4.14	1.447
weight	423	9.50	40.00	18.0863	4.68258
Dexmedetomidine bolus (mcg/Kg)	423	0	2	1.23	0.941
Remifentanil dose (Kg/min)	423	0.00	0.50	0.1012	0.14,428
Rocuronium dose (mg/Kg)	423	0.3	2.0	0.579	0.1304
Dexamethasone	423	0.000	1.500	0.29,722	0.090,514
Tramadol (mg/Kg)	423	0	2	0.27	0.469
Fluid (ml/Kg)	423	6.0	25.0	15.071	3.3976
Extubation time (min)	423	0	30	3.62	2.406
Surgery duration (min)	423	4	50	19.74	7.212
Time for a complete awakening	423	5	65	26.94	10.770

**Table 3:** Results from correlation analysis. For the chi-square test, the correlation factor phi is shown. For point–biserial correlation, Pearson’s R-value is shown as a correlation factor.

Variables	Type	p-value	Type of correlation	Correlation Factor
Age	Continuous	0.446	Point-biserial	
Gender	Categorical M (0)/F(1)		Chi-square	
ASA risk scale	Continuous		Point-biserial	
Pain during discharge	Categorical No (0), Yes (1)	<0.001	Chi-square	−0.202
PONV	Categorical No (0), Yes (1)	<0.001	Chi-square	−0.506
Oxygen desaturation during discharge	Categorical No (0), Yes (1)	0.016	Chi-square	−0.117
Other adverse events	Categorical No (0), Yes (1)	<0.001	Chi-square	−0.169
Fentanyl	Categorical No (0), Yes (1)	<0.001	Chi-square	−0.214
Dexmedetomidine continuous infusion	Categorical No (0), Yes (1)	<0.001	Chi-square	−0.355
Pain (FLACC)	Continuous	0.005	Point-biserial	−0.135
Extubation time	Continuous	0.002	Point-biserial	−0,137
Dexmedetomidine (Bolus)	Continuous	0.056	Point-biserial	
Administration time (induction)	Continuous	0.338	Point-biserial	
Remifentanil	Continuous	0.483	Point-biserial	
Rocuronium	Continuous	0.489	Point-biserial	
Dexamethasone	Continuous	0.361	Point-biserial	
Fluid therapy (intraoperative)	Continuous	0.598	Point-biserial	
Tramadol	Continuous	0.192	Point-biserial	
Surgery Duration	Continuous	0.514	Point-biserial	
Intellectual disability	Categorical No (0), Yes (1)	0.522	Chi-square	
Cardiovascular drug	Categorical No (0), Yes (1)	0.734	Chi-square	
Neuropsychiatric therapy	Categorical No (0), Yes (1)	0.734	Chi-square	
Chronic diseases	Categorical No (0), Yes (1)	0.889	Chi-square	
Infections 7 days preoperatively	Categorical No (0), Yes (1)	0.639	Chi-square	
Obstructive apnea syndrome	Categorical No (0), Yes (1)	0.723	Chi-square	
Preanesthesia	Categorical No (0), Yes (1)	0.641	Chi-square	
Midazolam	Categorical No (0), Yes (1)	0.397	Chi-square	
Ketamine	Categorical No (0), Yes (1)	0.317	Chi-square	
Parents at anesthesia induction	Categorical No (0), Yes (1)	0.067	Chi-square	
Intravenous anesthesia induction	Categorical No (0), Yes (1)	0.399	Chi-square	
Inhalational anesthesia induction	Categorical No (0), Yes (1)	0.420	Chi-square	
Type of inhalational agent sevoflurane-nitroxide	Categorical	0.494	Chi-square	
Clonidine dichotomy	Categorical No (0), Yes (1)	0.590	Chi-square	
Anesthesia Type (balanced or TIVA)	Categorical No (0), Yes (1)	0.237	Chi-square	
Bradycardia	Categorical No (0), Yes (1)	0.652	Chi-square	
Tachycardia	Categorical No (0), Yes (1)	0.940	Chi-square	
Hypotension	Categorical No (0), Yes (1)	0.189	Chi-square	
Hypertension	Categorical No (0), Yes (1)	0.380	Chi-square	
Intraoperative movements	Categorical No (0), Yes (1)	0.775	Chi-square	
Type of Electric knife	Categorical No (0), Yes (1)	0.466	Chi-square	
Oxygen desaturation	Categorical No (0), Yes (1)	0..834	Chi-square	
Laryngospasm	Categorical No (0), Yes (1)	0.860	Chi-square	
Emergence Delirium	Categorical No (0), Yes (1)	0.650	Chi-square	
Pain	Categorical No (0), Yes (1)	0.530	Chi-square	
Bradycardia	Categorical No (0), Yes (1)	0.519	Chi-square	
Delirium (PAED scale)	Continuous	0.525	Point-biserial	
Pain (FLACC)	Continuous	0.294	Point-biserial	

**Table 4:** VIF analysis of input features.

Feature	Variance Inflation Factor
Pain During Discharge	6.976561
PONV	1.586723
Dex continuous infusion	1.582372
Extubation time min	3.206748
Fentanyl use	2.169976
Pain (FLACC)	6.740975
Oxygen desaturation	1.256870
Other adverse events	1.127327

**Table 5:** Performance metrics measured across all the cross-validation folds.

	Precision	Recall	F1 score	ROC-AUC	PR-AUC	Accuracy
Random Forest	0.9924	**0.9450**	**0.9662**	0.8690	0.9886	0.9408
Logistic Regression	**0.9976**	0.8725	0.9222	0.9065	0.9914	0.8777
RUSBoost	0.9951	0.9000	0.9422	0.8746	0.9882	0.9014
AdaBoost	0.9948	0.9425	0.9641	**0.9250**	**0.9951**	**0.9413**

Abbreviation: Area Under the Receiver Operating Characteristic Curve (AUC- ROC), Precision-recall- Area Under the Receiver Operating Characteristic Curve (PR-AUC).

**Table 6:** Feature importance scores.

	Random Forest	AdaBoost	RusBoost	Logistic Regression
PONV	0.39	0.12	0.40	1.66
Dexmedetomidine infusion	0.04	0.06	0.21	0.75
Extubation time min	0.20	0.42	0.14	0.09
Fentanyl use	0.21	0.17	0.12	0.90
Pain During Discharge	0.11	0.14	0.09	1.08
Other adverse events	0.01	0.0	0.01	0.03
Oxygen desaturation (dichotomy)	0.03	0.08	0.0	0.26
